# KDM1A Promotes Immunosuppression in Hepatocellular Carcinoma by Regulating PD-L1 through Demethylating MEF2D

**DOI:** 10.1155/2021/9965099

**Published:** 2021-07-01

**Authors:** Yonglan Wang, Kun Cao

**Affiliations:** ^1^Department of Gastrointestinal Endoscopy Room, Jingmen No. 1 People's Hospital, Hubei, China; ^2^Department of Gastroenterology, Jingmen No. 1 People's Hospital, 168 Xiangshan Road, Jingmen 448000, China

## Abstract

**Background:**

Immune checkpoint inhibitor therapy targeting antiprogrammed cell death-1 (anti-PD-1) or its ligand (anti-PD-L1) is effective in the treatment of some hepatocellular carcinomas (HCC). Hence, further identification of biological targets related to PD-L1 regulation in HCC is beneficial to improve the clinical efficacy of immunotherapy. Some HCC cells express lysine-specific demethylase 1A (KDM1A), which is implicated in the reduced survival time of patients. Here, we studied whether the level of PD-L1 and the immunosuppression are regulated by KDM1A and its miRNA in HCC cells.

**Methods:**

In the present study, we studied clinical data from The Cancer Genome Atlas (TCGA) database. We performed qPCR and western blotting assays to measure the expression level of genes of interest. PD-L1 expression was also analyzed by FACS. Clustered regularly interspaced short palindromic repeats (CRISPR)/Cas9 was used to generate gene knockout cells to investigate the relationships of genes of interest. We also developed a reporter gene assay (RGA) to explore the changes in T cell-induced antitumor immunity relative to PD-L1 expression in HCC cells. The binding between proteins and promoters or miRNAs and their target genes was explored by luciferase reporter assays.

**Results:**

The results showed that PD-L1 and KDM1A were increased in HCC patients and cells, and KDM1A promoted the expression of PD-L1 in HCC cells. Our findings showed that the enhancement of PD-L1 expression was not attributed to mitochondrial dysfunction caused by increases in KDM1A in HCC cells. Furthermore, we observed a lower level of MEF2D methylation in HCC cells than in normal human liver cells. Demethylated MEF2D could bind to the promoter of PD-L1 and activate its expression, while KDM1A interacted with MEF2D and acted as a demethylase to reduce its methylation. Moreover, a new miRNA, miR-329-3p, targeting KDM1A was found to regulate the PD-L1 expression profile in HCC cells. In the xenograft model, the tumors treated with miR-329-3p showed growth inhibition.

**Conclusions:**

Mechanistically, miR-329-3p inhibits tumor cellular immunosuppression and reinforces the response of tumor cells to T cell-induced cytotoxic effect by targeting KDM1A mRNA and downregulating its expression, which contributed to MEF2D demethylation and activation of PD-L1 expression.

## 1. Introduction

Liver cancer, which is a global health problem, is the sixth most frequent malignancy and the fourth leading cause of cancer-related death worldwide [1]. Hepatocellular carcinoma (HCC), accounting for 90% of liver cancer, is the third most common cause of death globally, with a 5-year survival rate of 18% [1, 2]. Notably, HCC is a kind of immunogenic liver injury influenced by tumor-infiltrating lymphocytes, and lymphocyte-mediated antitumor immunity is able to prevent HCC malignancy as well as progression [3].

Programmed cell death-ligand 1 (PD-L1), encoded by the *CD274* gene, is the ligand of programmed cell death-1 (PD-1) and exists in several cancers, and it has the ability to inhibit T cell activation [4]. Neutralizing antibodies against immune checkpoints, such as PD-L1 or PD-1, show great performance as therapies for many cancers [4]. Therefore, the identification of biological targets related to PD-L1 regulation in HCC is helpful to improve the clinical efficacy of immunotherapy. Thus, it is necessary to further understand the pathways controlling PD-L1 expression in HCC to heighten the efficacy of PD-L1/PD-1 blockade.

Lysine-specific demethylase 1A (KDM1A, also named LSD1) was the first discovered histone-specific demethylase and is well known because of its ability to catalyze lysine demethylation in a flavin adenine dinucleotide- (FAD-) dependent oxidative reaction [5]. KDM1A could demethylate H3K4me1/2 (Lys-4) and H3K9me1/2 (Lys-9), which means it acts as a coactivator or a corepressor depending on the context [6–8]. In addition to histones, KDM1A is also able to demethylate lysine residues in several nonhistone proteins, such as p53 [9], Dnmt1 [10], E2F1 [11], and MYPT1 [12]. Because of its ability to control such a wide range of proteins, KDM1A is associated with an expansive spectrum of biological processes, such as cell proliferation [13], hematopoiesis [14], and embryonic development [15]. Evidence has shown that the dysregulation of KDM1A plays an important role in tumorigenesis in several cancers [12], including HCC. However, little is known about the relationship between KDM1A and T cell-mediated antitumor immunity in HCC.

MicroRNAs (miRNAs) are small noncoding RNAs that regulate a number of biological processes posttranscriptionally by binding to the 3′ untranslated region (UTR) of target mRNAs and suppressing their translation or accelerating their degradation [16]. Many miRNAs have been proven to play an important role in cancer progression by regulating the epithelial-mesenchymal transition (EMT), energetic metabolism, tumor angiogenesis [17–20], and other processes.

Here, we revealed that KDM1A could control the PD-L1 expression level in HCC by demethylating myocyte enhancer factor 2D (MEF2D), a transcription factor promoting PD-L1 expression without methylation. We also found that miR-329-3p could repress PD-L1 expression by targeting and downregulating KDM1A mRNA.

## 2. Materials and Methods

### 2.1. Cell Culture

The normal human liver cell line L-O2, human HCC cell lines HepG2 and SMMC7721, and mouse HCC cell line H22 were purchased from Procell Life Science & Technology. The L-O2, SMMC7721, and H22 cell lines were cultured in RPMI-1640 (Gibco) containing 10% fetal bovine serum (FBS) (Gibco), 100 *μ*g/mL streptomycin, and 100 U/mL penicillin (Gibco). HepG2 cells were cultured in MEM (Gibco) containing 100 *μ*g/mL streptomycin, 100 U/mL penicillin, and 10% FBS. All cell lines were cultured at 37°C with 5% CO_2_. All cell lines were routinely tested for mycoplasma contamination and found to be negative.

### 2.2. Clustered Regularly Interspaced Short Palindromic Repeats (CRISPR)/Cas9-Generated KDM1A, PD-L1, or MEF2D Knockout Cell Lines

The Cas9-GFP protein and sgRNA were obtained from GenScript. All sgRNA sequences are shown in Table S1. The CRISPR/Cas9 system was used as described in a previous study [21]. Briefly, before transfection, SMMC7721 cells (3 × 10^4^ cells per well) were seeded in a 6-well plate. Then, Opti-MEM containing the Lipofectamine Cas9 Plus™ Reagent (Invitrogen) was used to transfect a mixture of 50 pmol Cas9-GFP protein and 50 pmol sgRNA into SMMC7721 cells, which were cultured for 2 days at 37°C. Single clones were generated with the limiting dilution method in 96-well plates after sorting for GFP-positive cells by using a FACSAria instrument (BD Biosciences). All of the single clones were identified by sequencing and western blotting analyses. Cells with the successful KDM1A knockout (SMMC7721^KDM1A-/-^), PD-L1 knockout (SMMC7721^PD-L1-/-^), and MEF2D knockout (SMMC7721^MEF2D-/-^) were used for subsequent experiments.

### 2.3. Transduction and Transfection

SMMC7721^KDM1A-/-^ cells with stable KDM1A overexpression, SMMC7721^PD-L1-/-^ cells with stable PD-L1 overexpression, and SMMC7721^MEF2D-/-^ cells with stable overexpressing wild-type MEF2D (MEF2D-WT) or with K116R, K245R, K250R, and K279R mutation (MEF2D-4KR) were generated by transducing lentiviruses containing KDM1A, PD-L1, MEF2D-WT, and MEF2D-4KR, respectively. Briefly, 3 × 10^4^ cells were seeded into each well of 6-well plates with a 2 mL medium and cultured overnight. Then, cells were transduced with a 1 mL medium containing a 25 *μ*L lentiviral vector (4 × 10^8^ TU/mL) and 5 *μ*g/mL polybrene. 24 hours later, the cells were constantly maintained for another 24 h after changing the medium. Then, 6 *μ*g/mL puromycin was used to screen the transduced cells. The expression of proteins was confirmed by western blotting. SMMC7721 cells with stable overexpression of miR-329-3p or mi-NC (SMMC7721^miRNA^ or SMMC7721^mi-NC^) were also constructed with a lentivirus and screened with 6 *μ*g/mL puromycin.

The miR-329-3p inhibitor (inhibitor) and negative inhibitor control (inhibitor NC) were generated by RiboBio. For plasmid transfection, pcDNA3.1 (Invitrogen) was constructed and contained the target genes. Small interfering RNAs (siRNAs) were obtained from Sangon Biotech. Transfection using the Lipofectamine 2000 reagent (Thermo Fisher Scientific) was performed according to the standard protocol described in the manufacturer's instructions.

The sequences of miRNAs and inhibitors are shown in Table S1.

### 2.4. Antibodies and Reagents

The anti-PD-L1 antibody, anti-KDM1A antibody, and anti-MEF2D antibody were purchased from Abcam. Anti-GAPDH, anti-HA, and anti-Flag were purchased from Sino Biological. Anti-K267me was generated from Abmart by immunization with 263-APSR(meK)PDLR-271 [22]. ORY-1001 and rotenone were purchased from Sigma. All primers and sgRNAs are shown in Table S1.

### 2.5. Quantitative Real-Time Polymerase Chain Reaction (qRT-PCR)

The TRIzol reagent (Invitrogen) was used for extracting total RNA, and the PrimeScript RT reagent kit (TaKaRa) was used for synthesizing cDNAs except for those corresponding to the miRNA, which was generated with a TaqMan Advanced miRNA cDNA synthesis kit (Waltham). qPCR quantification was performed by using the TB Green® Fast qPCR Mix (TaKaRa) according to the manufacturer's manual on the ABI-7500 (Applied Biosystems). For testing miRNA expression, stem-loop qRT-PCR was used referring to others' work [23, 24]. Primers are shown in Table S1.

### 2.6. Western Blotting Analysis

Proteins were extracted from cells (RIPA, Beyotime Biotechnology) and tumors (One Step Animal Tissue Active Protein Extraction Kit, Sangon Biotech), and quantification of protein lysates was performed by a bicinchoninic acid assay kit (Boster). After that, the proteins were boiled for 5 min at 100°C in a loading buffer (20 *μ*g) and separated in an SDS-PAGE gel at 80 V for 30 min and then at 120 V for 1 h. After that, the proteins were electrotransferred into a nitrocellulose membrane (Boster) at 300 A for 1.5 h and blocked for 1 h with Tris-buffered saline containing 0.1% Tween 20 (TBST) and 5% fat-free milk. After that, the membranes were incubated with corresponding primary antibodies overnight at 4°C and secondary antibodies for 2 h at room temperature after washing. The membranes were measured by an image analysis system (Image-Pro Plus 6.0, Media Cybernetics) after incubating with a high-signal electrochemiluminescence kit (Fdbio Science).

### 2.7. Adenosine Triphosphate (ATP)/Adenosine Diphosphate (ADP) Measurements

The ATP/ADP level in cells was measured using bioluminescence detection (ADP/ATP Ratio Assay Kit, Abcam) according to the manufacturer's instructions. The cells were plated in 96-well microplates for 72 h. The bioluminescence intensities were measured on a multimode microplate reader (Synergy H1 Hybrid, BioTek).

### 2.8. Mitochondrial Membrane Potential Assay

The mitochondrial membrane potential was measured using fluorescence detection (JC-10 Assay Kit, Abcam) according to the manufacturer's instructions. The cells were cultured in 96-well, black-walled, clear-bottom plates and dyed with 50 mL of JC-10 solution. The fluorescence intensities (excitation/emission (Ex/Em) = 485/525 nm and Ex/Em = 540/590 nm) were measured on a multimode microplate reader (Synergy H1 Hybrid, BioTek).

### 2.9. Mitochondrial Reactive Oxygen Species (ROS) Measurement

Mitochondrial ROS was measured using a MitoSOX™ Red mitochondrial superoxide indicator (Invitrogen) according to the manufacturer's instructions. Cells were incubated with 5 *μ*M MitoSOX™ reagent working solution for 10 min at 37°C in the dark. After washing three times with warm PBS, we detected the ROS intensity with LSRFortessa SORP (BD Biosciences), and the data were analyzed in FlowJo.

### 2.10. Reporter Gene Assay (RGA) Procedure

Jurkat cells (American Type Culture Collection (ATCC)) were transduced with a lentivirus containing the luc2P/NFAT-RE/Hygro sequence from the pGL4.30[luc2P/NFAT-RE/Hygro] vector and were screened with 3 *μ*g/mL puromycin for 2 weeks to generate a stable cell pool. 2 × 10^4^ of SMMC7721 cells in a total volume of 100 *μ*L containing other reagents were seeded into one well of 96-well Costar plates. The medium change was performed 24 h after seeding, and 1 × 10^5^ engineered Jurkat cells in a 100 *μ*L medium containing the CD3 agonist antibody OKT3 (PeproTech) were added to the wells and incubated at 37°C with 5% CO_2_ for 6 h. Then, 100 *μ*L of the Promega Bio-Glo™ Luciferase Assay reagent was added, and the plate was subsequently shaken for 3 min on a titer plate shaker. The relative luciferase units (RLU) were finally determined by a GloMax®-Multi Detection or SpectraMax plate reader.

### 2.11. Flow Cytometry

The harvested cells were washed with phosphate-buffered saline (PBS) and incubated with antibodies in PBS containing 2% FBS for 60 min on ice. After washing, the samples were analyzed on an LSRFortessa SORP (BD Biosciences). The FACS data were analyzed by FlowJo. The FITC anti-PD-L1 and isotype were purchased from BioLegend.

### 2.12. Luciferase Reporter Assay

Luciferase reporter assays were carried out as described previously [25]. Briefly, cells seeded in 24-well plates were cotransfected with a 0.75 *μ*g vector expressing the MEF2D or control, 1.5 *μ*g PD-L1 promoter/luciferase reporter, and 0.2 *μ*g *β*-galactosidase reporter. 48 h later, the cells were lysed and the luciferase activity was detected by the Dual-Luciferase Reporter Assay System (Promega) on a Varioskan Flash instrument (Thermo Fisher Scientific) according to the manufacturer's instructions.

### 2.13. Immunoprecipitation (IP)

Cells were lysed in an NP-40 lysis buffer (50 mM Tris (pH 7.5), 150 mM NaCl, 1 mM EDTA, and 0.25% sodium deoxycholate) containing 1 mM phenylmethylsulfonyl fluoride (PMSF) (Sigma) and protease inhibitor cocktail (Sangon Biotech). Proteins were incubated with anti-MEF2D and Protein A/G PLUS-Agarose (Santa Cruz Biotechnology) at 4°C overnight. Then, the protein-bound beads were washed with a lysis buffer and denatured in an SDS buffer (0.1 M Tris-HCl (pH 6.8), 4% SDS, 20% glycerol, sodium pyrophosphate, 1 mM Na_3_VO_4_, and NaF) at 100°C for 10 min. Cell extracts and immunoprecipitated proteins were analyzed by SDS-PAGE.

### 2.14. Xenograft Model

H22 cells (1 × 10^6^) were injected into the left flank of each 6–8-week-old wild-type male BALB/c mouse (Guangdong Medical Laboratory Animal Center). For miR-329-3p or mi-NC intratumoral injection, mice with palpable tumors (~0.1 cm3) were randomly divided into two groups (6 mice/group) 14 days after H22 cell injection. Cholesterol-conjugated miR-329-3p or mi-NC (50 nmol) was intratumorally injected into the two groups three times per week for two weeks. Tumor growth was detected every 6 days. Tumors were weighed and isolated after mice were sacrificed at 6 weeks after grafting.

### 2.15. Chromatin Immunoprecipitation- (ChIP-) PCR Analysis

ChIP was performed using ChIP Kit-One Step (Abcam) according to the manufacturer's protocol. 2 *μ*L of antibodies was used in the ChIP process. Anti-MEF2D antibodies were the same in the IP assay. Anti-H3K4me1, anti-H3K4me2, anti-H3K4me3, anti-H3K9me1, anti-H3K9me2, and anti-H3K9me3 antibodies were obtained from Abcam. 2 *μ*L from 100 *μ*L input DNA and eluted DNA was detected by qPCR as described previously. The primers are shown in Table S1.

### 2.16. LDH-Based T Cell Killing Assay (Cytotoxicity Assay)

1 × 10^4^ target cells (SMMC7721) were seeded in triplicate in a 96-well U-bottom plate with an effector (human peripheral blood mononuclear cell (PBMC)) (SailiBio, Inc.) at a ratio of 10 : 1 (effector : target (E : T)) and coincubated in 200 *μ*L of RPMI-1640 containing 4% FBS for 6 h at 37°C with 5% CO_2_. The cytotoxicity was measured by the lactate dehydrogenase (LDH) release assay (Non-Radioactive Cytotoxicity Assay, Promega). The absorbance at 490 nm was recorded by using a microplate reader (BioTek Instruments, Inc., USA). Except for wells with E+T (samples), other wells were designed for controls: wells only with a medium (blank), wells only with PBMC (E only), and wells only with target cells (T only). Each control well was triplicated. The T only wells were completely lysed by adding 30 *μ*L lysis solution (10x). The cytotoxic rate % was calculated according to the following formula: cytotoxic rate% = (samples − E only − blank)/(T only − blank)∗100.

Cell-free supernatants from T cell and tumor cell coculture were harvested at 6 h for the presence of interleukin-2 (IL-2) and interferon-*γ* (IFN-*γ*). These cytokines were quantified by ELISA kits (Boster, China). All samples were tested in duplicate according to the manufacturer's instructions.

### 2.17. Statistics

All quantitative data were evaluated to determine the normality of the distribution using the Shapiro-Wilk test. Student's *t*-test was performed using Prism (version 5; GraphPad Software). To compare multiple groups, one-way ANOVA followed by Tukey's multiple comparison test was performed using Prism. The data are presented as the means ± SD. *p* < 0.05 was considered to indicate a statistically significant difference.

## 3. Results

### 3.1. KDM1A Controls the Level of PD-L1 in HCC

**Figure 1:**
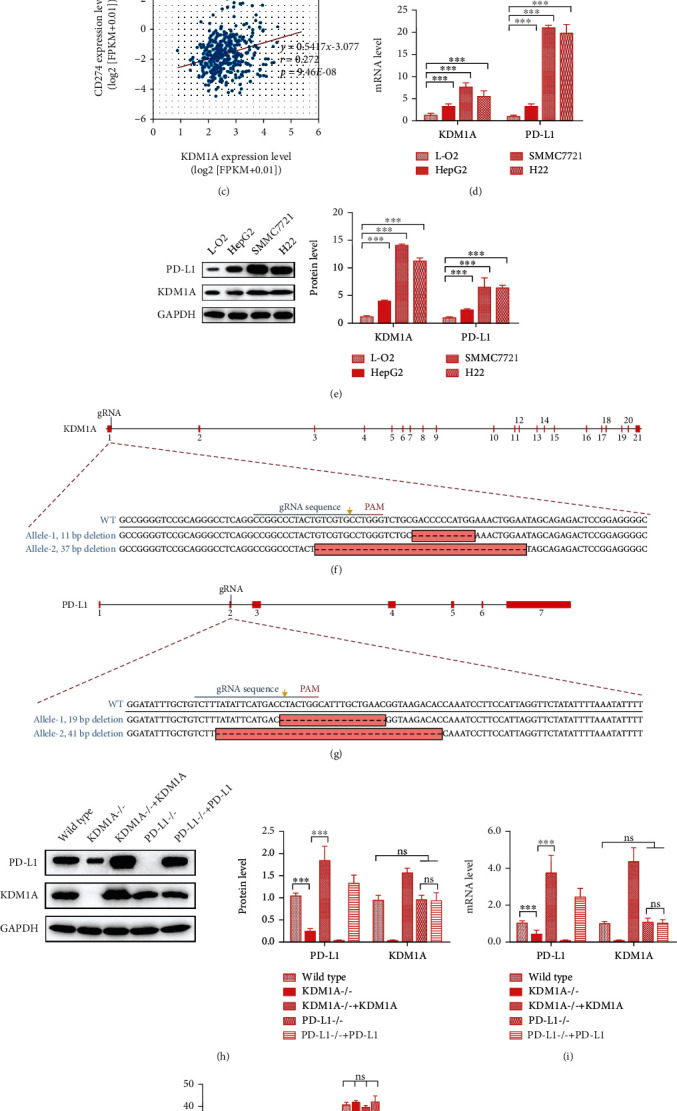
KDM1A increases the expression of PD-L1 in HCC cells. (a–c) Analysis of the clinical data of HCC patients from TCGA database. (a) The expression profile of KDM1A. (b) Association with overall survival of KDM1A. (c) Coexpression relationship of KDM1A and PD-L1. (d, e) mRNA expression (d) and protein expression (e) of KDM1A and PD-L1 in different cell lines. (f, g) Scheme of the gene type after the KDM1A (f) and PD-L1 (g) knockout in SMMC7721 cells. (h, i) Protein expression (h) and mRNA expression (i) of KDM1A and PD-L1 in wild-type SMMC7721 cells, SMMC7721^KDM1A-/-^ cells, SMMC7721^KDM1A-/-^ cells with KDM1A overexpression, and SMMC7721^PD-L1-/-^ cells, and SMMC7721^PD-L1-/-^ cells with PD-L1 overexpression. (j) ChIP-qPCR analysis of the PD-L1 promoter binding with H3K4me1/2/3 and H3K9me1/2/3 in wild-type SMMC7721 cells treated with the si-KDM1A or KDM1A expression plasmid. The data are presented as the means ± SD. *n* = 3 experiments in (a–i). ^∗^*p* < 0.05, ^∗∗^*p* < 0.01, and ^∗∗∗^*p* < 0.01.

First, we analyzed KDM1A expression in data available from TCGA database with the ENCORI website (http://starbase.sysu.edu.cn) to evaluate the importance of KDM1A in HCC. We observed a 2-fold higher level of KDM1A in HCC patients compared with healthy people in the clinic (*p* < 0.05; Figure 1(a)). The importance of KDM1A was also emphasized by its performance based on the overall survival curve for HCC, which demonstrated a significant difference in clinical outcomes between HCC patients and healthy persons (Figure 1(b)). In addition, the clinical coexpression analysis performed using the ENCORI website indicated that a high level of PD-L1 was related to high KDM1A expression, with a *p* value = 9.46*E* − 08 in HCC (Figure 1(c)). These findings implied that KDM1A may be involved in PD-L1 regulation and contribute to immunosuppression in HCC.

Based on the results of the clinical data analyses, we hypothesized that KDM1A could regulate the PD-L1 in HCC. Thus, we detected PD-L1 and KDM1A expression in different cell types, including the normal human liver cell line L-O2, the human HCC cell lines HepG2 and SMMC7721, and the mouse HCC cell line H22. KDM1A expression, as determined by qPCR and western blotting, was much higher in HCC cells than in normal human liver cells, which is similar to the tendency of PD-L1 expression observed in those cells (Figures 1(d) and 1(e)). Based on PD-L1 expression, we used SMMC7721 as our cell model in the *in vitro* assays.

To investigate the function of KDM1A in PD-L1 regulation, we generated a KDM1A knockout SMMC7721 cell line (named SMMC7721^KDM1A-/-^) and a PD-L1 knockout SMMC7721 cell line (named SMMC7721^PD-L1-/-^) using the CRISPR/Cas9 system. Both were derived from monoclones with genotype identification (Figures 1(f) and 1(g)). The detection of mRNA and protein expression showed that the knockout of the targeted gene in each cell line was successful (Figures 1(h) and 1(i)). In SMMC7721^KDM1A-/-^, PD-L1 expression was decreased, whereas KDM1A expression demonstrated no significant difference in SMMC7721^PD-L1-/-^ cells (Figures 1(h) and 1(i)). Moreover, we overexpressed KDM1A in SMMC7721^KDM1A-/-^ and PD-L1 in SMMC7721^PD-L1-/-^ cells to further explore the relationship between the two proteins. Interestingly, PD-L1 was elevated dramatically in SMMC7721^KDM1A-/-^ cells overexpressing KDM1A, while KDM1A exhibited a steady state in SMMC7721^PD-L1-/-^ cells regardless of the change in PD-L1 expression (Figures 1(h) and 1(i)).

Moreover, we want to know whether the PD-L1 expression variation was attributed to its chromosome openness affected by KDM1A, as it is a well-known histone demethylase. KDM1A can demethylate both “Lys-4” (H3K4me) and “Lys-9” (H3K9me) of histone H3 to control the H3K4me1/2/3 and H3K9me1/2/3 levels, which are associated with the chromosome openness [26]. Thereby, we have performed ChIP-qPCR to investigate this question. We measured the level of the PD-L1 promoter immunoprecipitated with anti-H3K4me1/2/3 or anti-H3K9me1/2/3 antibodies in wild-type SMMC7721 treated with the si-KDM1A or KDM1A overexpression plasmid. The results showed that the level of the PD-L1 promoter did not display a significant change responding to the KDM1A variation, indicating that KDM1A failed to affect the chromosome openness of PD-L1 directly (Figure 1(j)).

In general, these results suggested that KDM1A promotes the expression of PD-L1 independent with H3K4 and H3K9 demethylation in HCC.

### 3.2. The Alteration of PD-L1 Expression Is Irrelevant to Mitochondrial Dysfunction Caused by KDM1A in HCC

**Figure 2:**
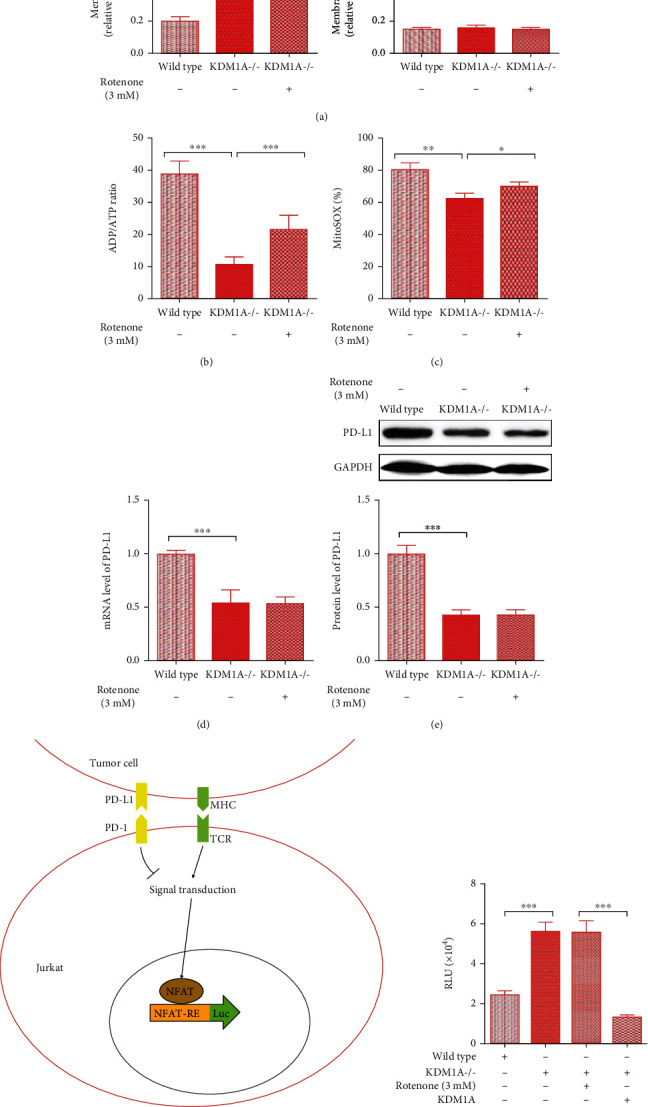
Mitochondrial dysfunction caused by elevated KDM1A does not affect the high level of PD-L1 in HCC cells. (a–c) The relative ratio of the JC-10 FL_590_/FL_525_ fluorescence intensity for mitochondrial membrane potential analysis (a), ATP/ADP analysis to determine ATP synthesis efficiency (b), and MitoSOX Red analysis to determine the mitochondrial ROS level (c) in wild-type SMMC7721 cells, SMMC7721^KDM1A-/-^ cells, and SMMC7721^KDM1A-/-^ cells with 3 mM rotenone treatment. (d, e) mRNA expression (d) and protein expression (e) of KDM1A and PD-L1 in the abovementioned groups. (f) Scheme of the principle of the RGA experiment. Jurkat cells have a TCR, which is responsible for recognizing tumor cells and activating the NFAT pathway. The strength of T cell-mediated antitumor immunity, which could be inhibited by the binding of PD-1 and PD-L1, is displayed by the luc2P/NFAT-RE elements in the engineered Jurkat cells. (g) The results of RGA reflecting the strength of T cell-mediated antitumor immunity in wild-type SMMC7721 cells, SMMC7721^KDM1A-/-^ cells, SMMC7721^KDM1A-/-^ cells treated with 3 mM rotenone, and SMMC7721^KDM1A-/-^ cells overexpressing KDM1A. The data are presented as the means ± SD. *n* = 3 experiments in (a–g). ^∗^*p* < 0.05, ^∗∗^*p* < 0.01, and ^∗∗∗^*p* < 0.01.

Several reports have implied that PD-L1 expression is connected with mitochondrial function to some extent in cancers, and KDM1A injures mitochondrial function in cancers [27–29]. Thus, it was necessary to inquire whether the regulation of the PD-L1 level was attributed to the mitochondrial function change caused by KDM1A. First, we used SMMC7721^KDM1A-/-^ cells to confirm mitochondrial function. The mitochondrial membrane potential (ΔΨm) is a key indicator for evaluating mitochondrial function. It was obvious that the knockout of KDM1A restored the ΔΨm in SMMC7721, as shown by the increase in the relative ratio of FL_590_/FL_525_, which is a value used to assess the level of ΔΨm (Figure 2(a)). Another key indicator is the efficiency of ATP synthesis, which is driven by ΔΨm and indicated by the relative ratio of ADP/ATP. The ATP synthesis efficiency was more robust in SMMC7721^KDM1A-/-^ cells with a lower ADP/ATP ratio than SMMC7721 cells (Figure 2(b)). We also detected ROS in cells by staining with MitoSOX. A lower percentage of MitoSOX-positive cells resulted in a decreased ROS level in SMMC7721^KDM1A-/-^ (Figure 2(c)). These results suggest that abolishing KDM1A rescues mitochondrial function in SMMC7721 cells.

Furthermore, we studied the relationship between PD-L1 expression and mitochondrial dysfunction by using rotenone to inhibit mitochondrial function in SMMC7721^KDM1A-/-^ cells. SMMC7721^KDM1A-/-^ cells treated with 3 mM rotenone exhibited a change in the ΔΨm, decreased ATP synthesis, and increased ROS levels, indicating that mitochondrial function was impaired (Figures 2(a)–2(c)). However, there was no significant difference in PD-L1 levels between the groups with or without rotenone treatment for SMMC7721^KDM1A-/-^ cells, which indicated that mitochondrial dysfunction did not contribute to PD-L1 regulation in HCC (Figures 2(d) and 2(e)).

In addition to PD-L1 expression, we also determined the ability of SMMC7721-based cell lines to inhibit T cell-mediated antitumor immunity through a RGA. This was based on the stable expression of a luciferase reporter under the control of the nuclear factor of activated T cell (NFAT) response elements in Jurkat cells (Figure 2(f)). The Jurkat cell line is an acute lymphocytic leukemia cell line derived from T lymphocytes, which can be activated by APCs or tumor cells with the CD3 agonist antibody OKT3 in T cell-like pathways, and this activation can be inhibited by immune checkpoints. In the Jurkat-based RGA, luciferase production only occurred in cells activated by SMMC7721 and OKT3, whereas the process was influenced by variation in the PD-L1 abundance on the SMMC7721 surface. Obviously, rotenone-induced mitochondrial dysfunction failed to decrease luciferase production in SMMC7721^KDM1A-/-^ cells (Figure 2(g)). In contrast, the groups of SMMC7721^KDM1A-/-^ overexpressing KDM1A showed a reduced luciferin signal compared to the SMMC7721 group (Figure 2(g)).

Together, these findings revealed that the changes in the PD-L1 steady state in HCC are not associated with changes in mitochondrial function caused by KDM1A, suggesting that another mechanism is involved in PD-L1 expression regulated by KDM1A.

### 3.3. KDM1A Demethylates MEF2D to Improve the Expression of PD-L1 in HCC

**Figure 3:**
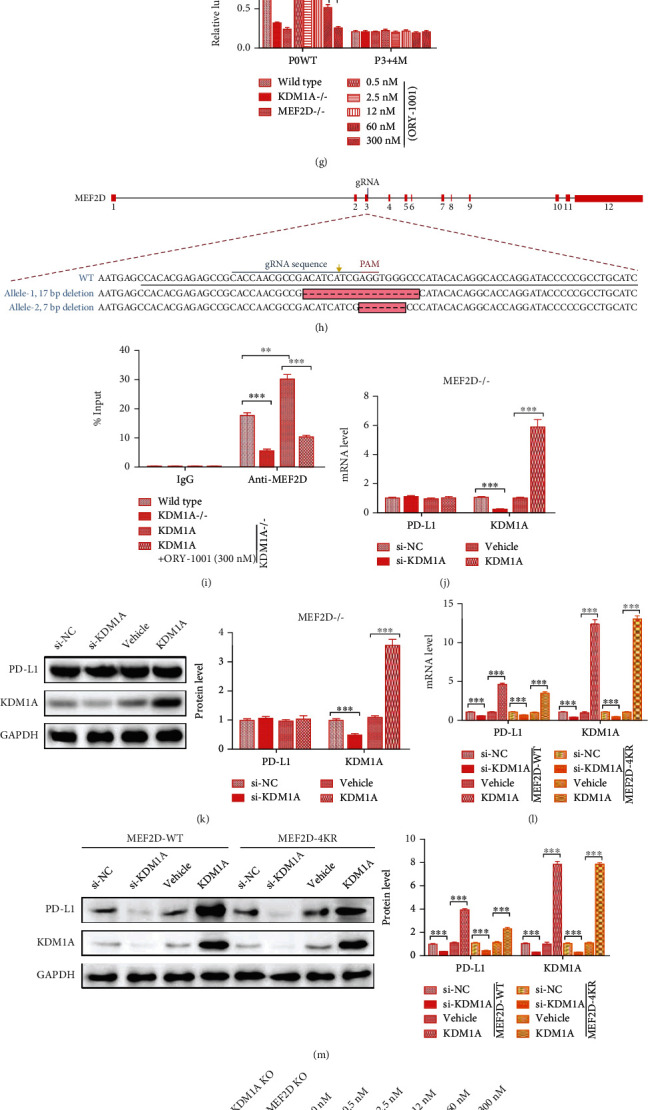
KDM1A binds to MEF2D, reduces its methylation, and leads to increased expression of PD-L1 in HCC cells. (a) The expression profile of MEF2D according to the clinical data of HCC patients from TCGA database. (b) 293T cells transiently expressed HA-MEF2D and Flag-KDM1A. HA-MEF2D was immunoprecipitated with anti-K267me, followed by immunoblotting with anti-HA and anti-Flag. (c) Top: proteins immunoprecipitated with anti-K267me were analyzed using anti-MEF2D in L-O2 cells and SMMC7721 cells. Bottom: protein expression in whole cell lysates of L-O2 cells and SMMC7721 cells. (d) mRNA expression of MEF2D in L-O2 cells and SMMC7721 cells. (e) Top: proteins immunoprecipitated with anti-K267me were analyzed using anti-MEF2D in SMMC7721^KDM1A-/-^ cells with or without KDM1A overexpression. Bottom: protein expression in whole cell lysates of SMMC7721^KDM1A-/-^ cells with or without KDM1A overexpression. (f) Scheme and sequences of the CD274 promoter reporter vectors. (g) Luciferase activity of CD274 promoter reporter vectors in wild-type SMMC7721 cells, SMMC7721^KDM1A-/-^ cells, SMMC7721^MEF2D-/-^ cells, and wild-type SMMC7721 cells treated with different concentrations of ORY-1001. (h) Scheme of the gene type after the MEF2D knockout in SMMC7721 cells. (i) ChIP-qPCR analysis of the PD-L1 promoter binding with MEF2D in wild-type SMMC7721 cells, SMMC7721^KDM1A-/-^ cells, SMMC7721^KDM1A-/-^ cells overexpressing KDM1A, and SMMC7721^KDM1A-/-^ cells overexpressing KDM1A and treating with 300 mM ORY-1001. (j, k) mRNA level (j) and protein level (k) of PD-L1 and KDM1A in SMMC7721^MEF2D-/-^ cells transfected with the si-NC, si-KDM1A, empty plasmid (vehicle), and KDM1A overexpression plasmid for 72 h. (l, m) mRNA level (l) and protein level (m) of PD-L1 and KDM1A in SMMC7721^MEF2D-/-+MEF2D-WT^ cells and SMMC7721^MEF2D-/-+MEF2D-4KR^ cells transfected with the si-NC, si-KDM1A, vehicle, and KDM1A overexpression plasmid for 72 h. (n) Top: proteins immunoprecipitated with anti-K267me were analyzed using anti-MEF2D. Bottom: protein expression in whole cell lysates. SMMC7721^KDM1A-/-^ cells, SMMC7721^MEF2D-/-^ cells, and wild-type SMMC7721 cells treated with different concentrations of ORY-1001. (o) FACS results for PD-L1 expression in wild-type SMMC7721 cells treated with different concentrations of ORY-1001. (p) The results of RGA reflecting the strength of T cell-mediated antitumor immunity in wild-type SMMC7721 cells, SMMC7721^KDM1A-/-^ cells, SMMC7721^MEF2D-/-^ cells, and wild-type SMMC7721 cells treated with different concentrations of ORY-1001. (q–s) The results of the cytotoxic rate, IFN-*γ* level, and IL-2 level in wild-type SMMC7721 cells, SMMC7721^KDM1A-/-^ cells, SMMC7721^MEF2D-/-^ cells, and wild-type SMMC7721 cells treated with different concentrations of ORY-1001. The data are presented as the means ± SD. *n* = 3 experiments in (a–o). ^∗^*p* < 0.05, ^∗∗^*p* < 0.01, and ^∗∗∗^*p* < 0.01.

Inspired by the novel findings that MEF2D regulates PD-L1 expression by its acetylation in HCC [25], we thought that MEF2D might play a significant role in the regulation of PD-L1 by KDM1A. We also found that the clinical outcome analysis showed an increased level of MEF2D in HCC patients (Figure 3(a)). Therefore, we adopted an IP assay to determine whether KDM1A could cause the demethylation of MEF2D. The anti-K267me antibody was used to identify the MEF2D methylation regulated by KDM1A [22]. Flag-KDM1A and HA-MEF2D recombinant proteins were cotransfected into 293T cells, and the methylation level in MEF2D was detected by western blotting. The results showed a reduction in MEF2D methylation in the presence of KDM1A, suggesting that KDM1A could induce MEF2D demethylation (Figure 3(b)).

Compared with that in L-O2 cells, MEF2D expression was higher in SMMC7721 cells, which was consistent with the clinical data (Figures 3(c) and 3(d)). However, the methylation of MEF2D was reduced by half in SMMC7721 cells, as analyzed by IP (Figure 3(c)). We next evaluated the MEF2D methylation level in SMMC7721^KDM1A-/-^ cells with or without KDM1A overexpression. The MEF2D methylation level decreased dramatically with KDM1A overexpression together with elevated PD-L1 expression (Figure 3(e)). The level of MEF2D displayed a slight increase, which may compensate for the change in its methylation and PD-L1 expression (Figure 3(e)). These results implied that the upregulation of PD-L1 expression caused by KDM1A occurred via demethylation of MEF2D in HCC.

Xiang et al. found that the binding sites of MEF2D were located in the -535 to -516 (P3) and -246 to -245 (P4) positions of the PD-L1 promoter [25]. According to the results, we used a reporter gene assay to investigate the influence of MEF2D methylation on its ability to bind to the PD-L1 promoter (Figure 3(f)). The reporter vectors consisted of a luciferase gene activated by the wild-type PD-L1 promoter (P0WT) and mutant PD-L1 promoter (P3+4M) that were transfected into different cells. The demethylation of MEF2D caused by KDM1A was inhibited by ORY-1001, a micromolecule inhibiting the demethylase activity of KDM1A. It was obvious that the luciferase activity was decreased in SMMC7721 cells with KDM1A or MEF2D abolishment (Figure 3(g)). In wild-type SMMC7721 cells with the P0WT promoter, the reduction in luciferase activity exhibited a dose-dependent effect with ORY-1001 (Figure 3(g)). SMMC7721 cells without MEF2D were generated by CRISPR/Cas9, similar to the SMMC7721^KDM1A-/-^ cells, with 17 bp and 7 bp deletions within the alleles, respectively (Figure 3(h)). Then, we measured the binding between the MEF2D and the PD-L1 promoter in different conditions by ChIP-qPCR. The results showed a decreased enrichment of PD-L1 promoter tendency along with the KDM1A knockout or activity inhabitation, while its enrichment increased with KDM1A overexpression (Figure 3(i)). We also found that the PD-L1 level kept a stable state in SMMC7721^MEF2D-/-^ cells treated with si-KDM6A or KDM6A overexpression (Figures 3(j) and 3(k)).

Besides, Xiang et al. have found that p300 could promote PD-L1 expression through its acetylation activity on K116, K245, K250, K267, and K279 in MEF2D, while SIRT7 could inhibit PD-L1 expression by deacetylating those lysines [25]. p300 suggested K116 as a major acetylation site and K245, K250, K267, and K279 as minor acetylation sites. Thereby, we constructed SMMC7721^MEF2D-/-^ cell lines overexpressing MEF2D-WT or MEF2D-4KR, similar to Xiang et al.'s work [25]. We test the acetylation of MEF2D-4KR induced by p300 and found a few acetylation levels with no effect on PD-L1 expression compared to MEF2D-WT (data were not shown). Then, we treated SMMC7721^MEF2D-/-+MEF2D-WT^ or SMMC7721^MEF2D-/-+MEF2D-4KR^ with the KDM1A knockdown or overexpression. The western blotting and qPCR results showed a significant change in PD-L1 expression associated with the KDM1A level in SMMC7721^MEF2D-/-+MEF2D-4KR^ cells, compared with SMMC7721^MEF2D-/-+MEF2D-WT^ cells (Figures 3(l) and 3(m)), excluding the possibility of MEF2D in regulating PD-L1 independent with its demethylation induced by KDM1A. These results indicated that a higher level of KDM1A-induced MEF2D demethylation triggers more intense binding between the MEF2D and the PD-L1 promoter.

Moreover, we investigated the influence of KDM1A activity on immunosuppression in SMMC7721 cells. We performed a FACS assay to monitor the PD-L1 level on the surface of SMMC7721 cells treated with different concentrations of ORY-1001. ORY-1001 reduced PD-L1 abundance at the cellular surface associated with the inhibited MEF2D methylation level (Figures 3(n) and 3(o)). Consistent with the FACS results, the luciferase activity showed an ORY-1001 dose-dependent tendency, with the highest signal observed in KDM1A- or MEF2D-lacking cells and the lowest signal observed in wild-type cells (Figure 3(p)). Similarly, the LDH-based T cell killing assay also displayed a progressive cytotoxic rate, as well as the level of IFN-*γ* and IL-2, with the higher ORY-1001 concentration (Figures 3(q)–3(s)). These results suggested that the suppression of KDM1A activity subdued the immunosuppression of SMMC7721 cells and promoted the T cell response.

Together, these data demonstrated that KDM1A-driven MEF2D demethylation promotes PD-L1 abundance and plays a suppressive role in T cell-mediated antitumor immunity in HCC.

### 3.4. hsa-miR-329-3p Is a PD-L1 Inhibitor That Targets KDM1A and Downregulates Its Expression in HCC

Considering the significant influence of miRNAs on cancer progression, we started to investigate whether there was a miRNA regulating PD-L1 expression by binding to KDM1A. To explore potential miRNAs targeting KDM1A, we predicted possible miRNAs with the ENCORI website, including several prediction databases, such as PITA, RNA22, miRmap, microT, miRanda, PicTar, and TargetScan. We found 5 miRNA candidates with predicted binding in at least 3 databases, including hsa-miR-28-5p, hsa-miR-137, hsa-miR-329-3p, hsa-miR-362-3p, and hsa-miR-708-5p (Table 1). Expression analysis in HCC patients based on data from TCGA database performed by ENCORI showed a significant difference with a *p* value < 0.05 except for hsa-miR-708-5p (Table 2). Coexpression analysis in HCC patients of miRNA candidates and KDM1A mRNA showed that only two miRNAs, hsa-miR-137 and hsa-miR-329-3p, displayed a correlation with KDM1A mRNA expression with a *p* value < 0.05 (Table 3). Consistently, hsa-miR-137 has been proven to target KDM1A mRNA in the progression of prostate cancer [30]. Thus, we assumed that hsa-miR-329-3p might be another miRNA targeting KDM1A in HCC.

**Figure 4:**
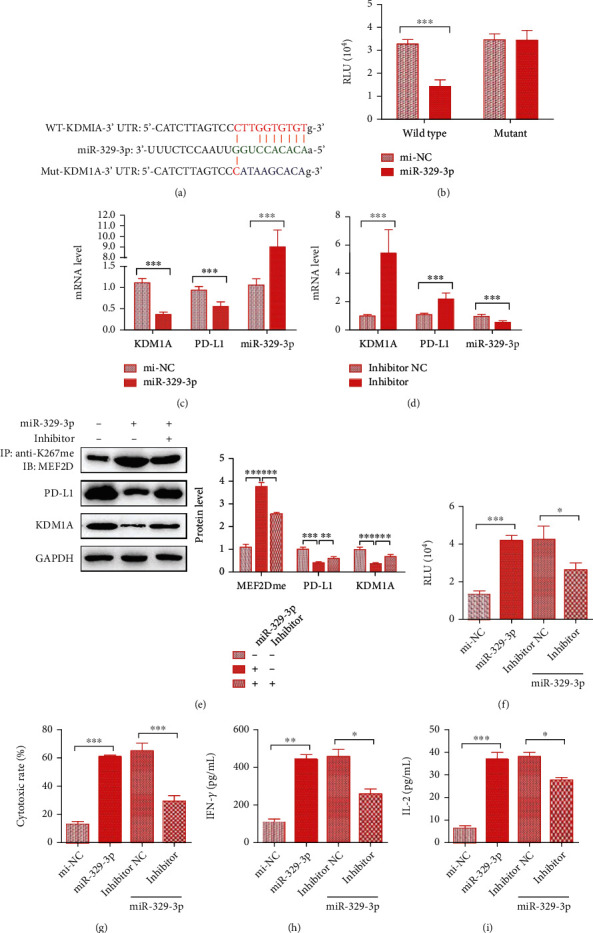
hsa-miR-329-3p inhibits PD-L1 expression by downregulating KDM1A levels in HCC cells. (a) The sequences of the KDM1A 3′ UTR wild type (WT), KDM1A 3′ UTR mutant (Mut), and miR-329-3p, and the schematic diagram of target prediction between KDM1A and miR-329-3p. (b) Binding of miR-329-3p to the 3′ UTR of KDM1A in HEK293T cells by luciferase reporter assay. (c, d) mRNA/miRNA expression of KDM1A, PD-L1, and miR-329-3p in SMMC7721^miRNA^ cells treated with the control (c) or in SMMC7721^miRNA^ cells treated with the inhibitor and control (d). (e) Top: proteins immunoprecipitated with anti-K267me were analyzed using anti-MEF2D. Bottom: protein expression in whole cell lysates from wild-type SMMC7221 cells, SMMC7721^miRNA^ cells, and SMMC7721^miRNA^ cells with the inhibitor. (f) The results of RGA reflecting the strength of T cell-mediated antitumor immunity in SMMC7221 cells stably overexpressing mi-NC, SMMC7721^miRNA^ cells, and SMMC7721^miRNA^ cells with the inhibitor or control. (g–i) The results of the cytotoxic rate, IFN-*γ* level, and IL-2 level in SMMC7221 cells stably overexpressing mi-NC, SMMC7721^miRNA^ cells, and SMMC7721^miRNA^ cells with the inhibitor or control. The data are presented as the means ± SD. *n* = 3 experiments in (a–i). ^∗^*p* < 0.05, ^∗∗^*p* < 0.01, and ^∗∗∗^*p* < 0.01.

To further confirm the predicted results for miR-329-3p, we performed a dual-luciferase reporter assay that utilized wild-type and mutant KDM1A 3′ UTR luciferase plasmids as well as miR-329-3p and mi-NC mimics according to the predicted alignment (Figures 4(a) and 4(b)) [31]. Cells cotransfected with the wild-type KDM1A 3′ UTR luciferase and miR-329-3p mimics showed a conspicuous reduction in luciferase activity relative to the cells cotransfected with the wild-type KDM6A 3′ UTR and mi-NC, while there was no difference in activity for cells with the mutant KDM1A 3′ UTR luciferase and miR-329-3p or mi-NC mimics, indicating that KDM1A mRNA is the target of miR-329-3p.

Then, we constructed SMMC7721 cells stably overexpressing miR-329-3p (SMMC7721^miRNA^) or mi-NC (SMMC7721^mi-NC^) and monitored the variation in KDM1A and PD-L1 expression. At the mRNA level, KDM1A and PD-L1 mRNA levels decreased dramatically, resulting in nearly 9-fold higher miR-329-3p expression (Figure 4(c)) compared with that in the mi-NC groups. In contrast, SMMC7721^miRNA^ cells exhibited increased KDM1A and PD-L1 mRNA levels when transfected with the miR-329-3p inhibitor, which decreased miRNA expression (Figure 4(d)). Consistent with the mRNA results, miR-329-3p overexpression decreased KDM1A expression and further decreased PD-L1 levels, leading to the upregulation of MEF2D methylation, while the miR-329-3p inhibitor reversed this effect (Figure 4(e)). Furthermore, we detected the effect of miR-329-3p on the anti-immune activity of SMMC7721 cells by the Jurkat-based RGA. Relative to that in the SMMC7721^mi-NC^ group, the luciferase activity increased greatly in the SMMC7721^miRNA^ group, but it was suppressed by transfection with the miR-329-3p inhibitor (Figure 4(f)). What is more, an elevated level of the T cell-induced cytotoxic rate as well as IFN-*γ* and IL-2 release levels was observed in miR-329-3p transfected target cells, whereas all of them were repressed dramatically when treated with the miR-329-3p inhibitor (Figures 4(g)–4(i)).

Our findings suggested that hsa-miR-329-3p reduced the immunosuppression in HCC cells and promotes its response toward T cell-induced cytotoxic effect by inhibiting PD-L1 expression in HCC through targeting KDM1A mRNA and indirectly reducing MEF2D demethylation.

### 3.5. miR-329-3p Enhances the T Cell Response toward HCC Tumors by Controlling the Expression of PD-L1 in the KDM1A/MEF2D Pathway

**Figure 5:**
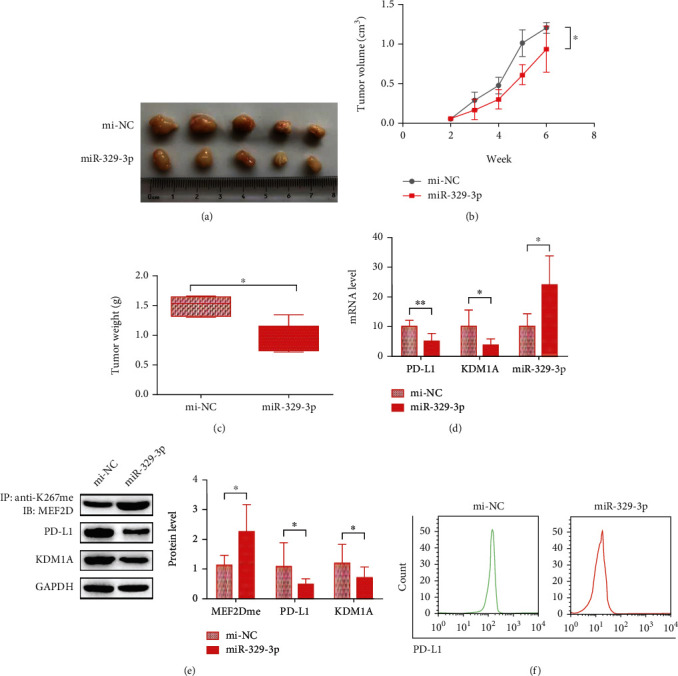
hsa-miR-329-3p inhibits HCC tumor growth by increasing the T cell response by controlling PD-L1 expression in the KDM1A/MEF2D pathway. (a–c) The volume and weight of subcutaneous xenograft tumors of H22 cells with or without miR-329-3p isolated from mice. The volume was measured every week starting at 2 weeks after injection. The weight was measured at 6 weeks after injection. (d) mRNA/miRNA expression of KDM1A, PD-L1, and miR-329-3p in xenograft tumors at 6 weeks after injection. (e) Top: proteins immunoprecipitated with anti-K267me were analyzed using anti-MEF2D. Bottom: protein expression in whole cell lysates from xenograft tumors at 6 weeks after injection. (f) FACS results of PD-L1 expression in xenograft tumors at 6 weeks after injection. The data are presented as the means ± SD. *n* = 6 experiments in (a–f). ^∗^*p* < 0.05, ^∗∗^*p* < 0.01, and ^∗∗∗^*p* < 0.01.

To study the impact of miR-329-3p on the immune response of HCC tumors, a wild-type BALB/c mouse xenograft model was developed by implanting H22 cells with high PD-L1 and KDM1A expression. After two weeks of cell implantation, we intratumorally injected cholesterol-conjugated mi-NC or miR-329-3p mimics into mice three times per week. It was easily observed that miR-329-3p prevented tumor growth, which was verified again by the tumor volume measurements (Figures 5(a) and 5(b)). Moreover, the tumor weight in the miR-329-3p group, which was measured at least weekly, was much lower than that in the mi-NC group (Figure 5(c)). These results suggested that miR-329-3p prevents HCC tumor growth *in vivo*.

Thus, we focused on the mechanism of HCC tumor growth inhibition by miR-329-3p. The mRNAs of KDM1A and PD-L1 as well as miR-329-3p in separated tumors at the end of 6 weeks were detected first. Compared with the tumors injected with mi-NC, the tumors injected with mi-329-3p displayed decreased expression of KDM1A and PD-L1 mRNA (Figure 5(d)). We also found similar results at the protein level, with lower expression of KDM1A and PD-L1 in miR-329-3p-injected tumors and elevated MEF2D methylation (Figure 5(e)). In addition, we tested the abundance of PD-L1 in tumor cells by using FACS, and the results indicated that miR-329-3p-injected tumors exhibited lower PD-L1 abundance than the mi-NC group tumors (Figure 5(f)).

Together, all the results showed that miR-329-3p inhibits HCC tumor growth by receding immunosuppression through downregulating KDM1A which results in PD-L1 reduction.

## 4. Discussion

An increasing number of studies have revealed the importance of KDM1A in cancer. On the one hand, KDM1A plays a role as a transcription cofactor in cancer progression. For example, disruption of the KDM1A-GFI interaction, which regulates gene expression, resulted in the promotion of blast cell differentiation in acute myeloid leukemia (AML) with malignant lymphoma with leukemia (MLL) translocations [32]. In another case, KDM1A interacted with Snail1 to target gene promoters and inhibit cell migration and invasion in metastasis [33]. On the other hand, the regulation of KDM1A in cancer progression is mediated by the demethylation of nonhistone proteins. The first identified nonhistone protein was the tumor suppressor p53, the transcriptional activity of which is suppressed to promote cancer progression. KDM1A also demethylates Ago2 at the K726me1 site to regulate the tumor T cell response [34]. In addition, KDM1A has been regarded as a potential target for anticancer drug development, as a number of reports indicate its high levels in many carcinomas, including prostate cancer, lung cancer, and breast cancer [35]. Accordingly, several KDM1A inhibitors are in clinical trials [5]. However, little is known about the relationship between KDM1A and immune checkpoints in cancer. In our study, we found that a higher level of PD-L1 was associated with elevated KDM1A expression in HCC cells, which was not previously reported.

Notably, KDM1A could induce mitochondrial dysfunction in HCC cells. For example, Sakamoto et al. found that KDM1A regulated glycolytic and mitochondrial metabolism in HCC and promoted its progression [29]. In this case, the oxygen consumption rate (OCR) was increased in the KDM1A knockdown HepG2 cells. In our study, it was confirmed that SMMC7721 cells with the KDM1A knockout exhibited increased mitochondrial activity. However, PD-L1 expression seems to be potentially related to mitochondrial dysfunction. AKT is the main protein that promotes metabolic reprogramming to switch from aerobic respiration to anaerobic glycolysis, whereas its inhibitors inhibit PD-L1 expression in cancer cells [27]. Thus, it was necessary to verify whether the increased PD-L1 abundance in SMMC7721 cells was attributed to mitochondrial dysfunction caused by KDM1A. Rotenone is a mitochondrial electron chain inhibitor. Yin et al. used it to inhibit mitochondrial function to reduce ROS in HCC cells, which exhibited a higher level of ROS in the control group [36]. We used rotenone to repress mitochondrial function, which was rescued by the KDM1A knockout in HCC cells, to investigate the relationship between mitochondrial dysfunction and PD-L1 expression. However, our results indicated that there was no connection between PD-L1 expression and changes in mitochondrial function.

MEF2D is a transcription factor and participates in many oncogenesis processes. MEF2D, for example, promotes epithelial-mesenchymal transition, metastasis, and angiogenesis in colorectal cancer [37, 38]. In HCC, a high level of MEF2D is associated with a poor prognosis [39]. MEF2D also supports or promotes cell proliferation in HCC cells and is required for tumorigenicity [39]. In a recent study, a new function of MEF2D was demonstrated in the regulation of PD-L1 expression in HCC via its acetylation or deacetylation, which is controlled by p300 and SIRT7 [25]. Interestingly, Choi et al. found that KDM1A could demethylate MEF2D and increase its transcriptional activity during skeletal muscle cell differentiation [22]. Hence, we explored the possibility of the regulation of PD-L1 by the KDM1A/MEF2D axis in HCC. We found that the reduction in MEF2D methylation caused by KDM1A dramatically increased its binding to the PD-L1 promoter and elevated PD-L1 expression in HCC, as confirmed by the use of the KDM1A inhibitor ORY-001. As a result, antitumor immunity mediated by T cells was also suppressed by decreased MEF2D methylation.

Many studies have shown that miRNAs profoundly affect HCC progression [40]. Hence, we investigated miRNAs targeting KDM1A and regulating PD-L1 expression in HCC. Guo and Wang revealed the role of miR-329-3p as a suppressor of HCC cell proliferation, invasion, migration, and EMT processes as well as tumor growth by downregulating FOXK1 and inhibiting the AKT/mTOR pathway [41]. However, in the present study, we found a different mechanism for the antitumor activity of miR-329-3p. We proved that miR-329-3p could target the 3′ UTR of KDM1A and regulate its steady state in HCC cells. We also determined the functional role of miR-329-3p as a PD-L1 inhibitor that could promote T cell-mediated antitumor immunity in HCC cells. Thus, miR-329-3p exerted antitumor activity by increasing T cell infiltration in xenograft mice. The different mechanisms revealed in the two studies emphasized the importance of miR-329-3p in HCC and implied that further research needs to be done to explore the role of miRNAs and carcinomas.

## 5. Conclusion

We demonstrated a new mechanism of PD-L1 regulation carried out by KDM1A-induced MEF2D demethylation. KDM1A promotes PD-L1 abundance and enhances immunosuppression activity in HCC by reducing MEF2D methylation. In contrast, miR-329-3p suppresses PD-L1 expression by targeting KDM1A and reinforces the response to T cell-induced cytotoxic effect in HCC cells.

## Figures and Tables

**Table 1:** The predictive results of miRNA targeting KDM1A.

miRNA name	PITA	RNA22	miRmap	microT	miRanda	PicTar	TargetScan	Num of database
hsa-miR-28-5p	1	0	1	0	1	0	0	3
hsa-miR-137	1	0	1	0	1	1	1	5
hsa-miR-329-3p	1	0	1	0	1	0	0	3
hsa-miR-362-3p	1	0	1	0	1	0	0	3
hsa-miR-708-5p	1	0	1	0	1	0	0	3

**Table 2:** Expression analysis of predicted miRNAs in HCC patients from TCGA database.

miRNA name	CancerNum	NormalNum	CancerExp	NormalExp	Fold change	*p* value	FDR
hsa-miR-28-5p	370	50	159.9	197.77	0.81	0.000003	0.000031
hsa-miR-137	370	50	1.33	0.06	20.82	0.014	0.056
hsa-miR-329-3p	370	50	1.84	0.95	1.94	0.017	0.066
hsa-miR-362-3p	370	50	4.27	2.26	1.89	8.7*E* − 09	1.5*E* − 07
hsa-miR-708-5p	370	50	3.14	1.8	1.75	0.061	0.19

**Table 3:** Coexpression analysis in HCC patients between miRNA candidates and KDM1A mRNA.

miRNA name	Coefficient *R*	*p* value
hsa-miR-28-5p	0.031	0.555
hsa-miR-137	0.369	2.19*E* − 13
hsa-miR-329-3p	0.231	7.28*E* − 06
hsa-miR-362-3p	0.032	0.546

## Data Availability

The data used to support the findings of this study are available from the corresponding author upon request.

## Abbreviations

HCC: Hepatocellular carcinoma
PD-L1: Programmed cell death-ligand 1
PD-1: Programmed cell death-1
KDM1A: Lysine-specific demethylase 1A
FAD: Flavin adenine dinucleotide
miRNAs: MicroRNAs
UTR: Untranslated region
EMT: Epithelial-mesenchymal transition
MEF2D: Myocyte enhancer factor 2D
FBS: Fetal bovine serum
CRISPR: Clustered regularly interspaced short palindromic repeats
qRT-PCR: Quantitative real-time polymerase chain reaction
TBST: Tris-buffered saline containing 0.1% Tween 20
ATP: Adenosine triphosphate
ADP: Adenosine diphosphate
ROS: Reactive oxygen species
RGA: Reporter gene assay
ATCC: American Type Culture Collection
RLU: Relative luciferase units
PBS: Phosphate-buffered saline
IP: Immunoprecipitation
PMSF: Phenylmethylsulfonyl fluoride
ΔΨm: Mitochondrial membrane potential
CCCP: Carbonyl cyanide 3-chlorophenylhydrazone
NFAT: Nuclear factor of activated T cell
DMSO: Dimethyl sulfoxide
AML: Acute myeloid leukemia
MLL: Malignant lymphoma with leukemia
OCR: Oxygen consumption rate
ChIP-qPCR: Chromatin immunoprecipitation- (ChIP-) PCR
IL-2: Interleukin-2
IFN-*γ*: Interferon-*γ*
PBMC: Peripheral blood mononuclear cell.

## Supplementary Materials

Supplementary Materials Table S1: the sequences of sgRNAs, primers, and miRNAs.
