# Supplementary material for: KDM1A Promotes Immunosuppression in Hepatocellular Carcinoma by Regulating PD-L1 through Demethylating MEF2D
Source: J Immunol Res. 2021 Jul 1;2021:9965099. doi: 10.1155/2021/9965099 (PMC8270703; doi:10.1155/2021/9965099)
Supplement: Supplementary Materials — Table S1: the sequences of sgRNAs, primers, and miRNAs. [file 9965099.f1.docx]

Table S1: The sequences of sgRNAs, primers and miRNA.

| Name | Application | Sequence |
| --- | --- | --- |
| KDM1A-sgRNA | knockout | CCGGCCCTACTGTCGTGCCT |
| CD274-sgRNA | knockout | TCTTTATATTCATGACCTAC |
| MEF2D-sgRNA | knockout | CACCAACGCCGACATCATCG |
| KDM1A KO TEST-F | PCR | AGCAGGCGGCTCCGAGAACGG |
| KDM1A KO TEST-R |  | CAACCGTAACCCCACGTCAGG |
| CD274 KO TEST-F | PCR | GGGCAATGGAATGAAGATATTGA |
| CD274 KO TEST-R |  | GGTGATGCCAGTACTGTGTTA |
| MEF2D KO TEST-F | PCR | GGCCTTAACTGAGCCAGATCTG |
| MEF2D KO TEST-R |  | CTTCAACAGTTCACAATGTTGGC |
| has-miR-329-3p-F | qPCR | GTGGAACAGACCTGGTAAAC |
| has-miR-329-3p-R |  | CAAGTGCGAGTCGTGCAGT |
| GAPDH-F | qPCR | GGAAGGACTCATGACCACAGTCC |
| GAPDH-R |  | TCGCTGTIGAAGTCAGAGGAGACC |
| KDM1A-F | qPCR | TGACCGGATGACTTCTCAAGA |
| KDM1A-R |  | GTTGGAGAGTAGCCTCAAATGTC |
| CD274-F | qPCR | TGGCATTTGCTGAACGCATTT |
| CD274-R |  | TGCAGCCAGGTCTAATTGTTTT |
| MEF2D-F | qPCR | CCAGCGAATCACCGACGAG |
| MEF2D-R |  | GCAGTCACATAGCACGCTC |
| CD274-p-F | ChIP-qPCR | TTGGGCCCATTCACTAACCC |
| CD274-p-R |  | AAGAACTTCCCATCCCGAGC |
| has-miR-329-3p | miRNA | UUUCUCCAAUUGGUCCACACAa |
| mi-NC |  | UCACAACCUCCUAGAAAGAGUAGA |
| si-NC | siRNA | UUCUCCGAACGUGUCACGU |
| si-KDM6A | siRNA | ACAGUAUCACUGUUAUAAGGU |
